# Formulation of traditional Chinese medicine and its application on intestinal flora of constipated rats

**DOI:** 10.1186/s12934-020-01473-3

**Published:** 2020-11-18

**Authors:** Sihan Li, Youcheng He, Haiou Zhang, Rong Zheng, Ruoying Xu, Qihong Liu, Shuihua Tang, Xiao Ke, Minghan Huang

**Affiliations:** 1 Department of Gastroenterology, The Second People’s Hospital affiliated to Fujian University of Traditional Chinese Medicine, Fuzhou, 353003 China; 2 School of Basic Medical Sciences, Guangzhou University of Chinese Medicine, Guangzhou, China

**Keywords:** Constipation, Traditional Chinese medicine, Gut microbiota, *Lactobacillus*, Firmicutes

## Abstract

In this study, the self-extracted constipation treatment of traditional Chinese medicine extracts was applied to constipated rats. To explore the mechanism and role of the Chinese medicine for the treatment of constipation, the 16S rRNA sequencing and qRT-PCR technology were used to analyze the intestinal flora. We found that the relative abundance of *Firmicutes* with constipation was significantly higher accounted for 86.7%, while the gut microbiota was significantly changed after taking a certain dose of Chinese medicine, greatly increased the relative abundance of *Lactobacillus* accounted for 23.1%, enhanced the symbiotic relationships of *Lactobacillus* with other intestinal flora*.* The total copies of intestinal bacteria in the constipated rats decreased after taking the traditional Chinese medicine. Finally, this study results provides a theoretical basis for the treatment and understand the mechanism and effect of traditional Chinese medicine on rate constipation.

## Introduction

Constipation is a common clinical gastrointestinal disease, mainly manifested as irregular defecation, difficulty in defecation. The total incidence rate of the disease is around 16% [1]. As the disease spectrum changes, Chronic constipation has gradually become a common problem affecting children's physical and mental health [2, 3]. According to a US survey, constipation plagues in healthy people, and constipation are also associated with colon cancer, heart, brain vessels, and the elderly dementia and so on [4]. Meanwhile, long term constipation can lead to intestinal wall damage, hemorrhoids and anal fissure, and the accumulation of enterotoxin can also easily lead to cytopathic changes, thus leading to colorectal cancer [5]; elderly constipation patients with cardiovascular and cerebrovascular diseases can induce acute myocardial infarction and other serious diseases due to excessive abdominal pressure due to forced defecation. In addition, constipation has an important relationship with hepatic encephalopathy, breast disease, Alzheimer’s disease, women’s dysmenorrhea, urinary tract infection and other diseases [4].

The pathogenesis of chronic constipation mainly includes the reduction of colonic contraction, the dysfunction of pelvic floor and defecation, the abnormality of intestinal neurons and neurotransmitters, and the abnormality of intestinal neurochemical signals [6]. In recent years, it has been found that the imbalance of gut microbiota plays an important role in the pathogenesis of chronic constipation. Intestinal microflora have an important functions in maintaining homeostasis and improving immunity [7]. However, if the feces of patients with constipation stay in the intestine for a long time, it may change the intestinal microflora, metabolic molecules of intestinal microflora, such as methane and short chain fatty acids. Between the microflora and the host immune system plays an important role in the pathogenesis of chronic constipation [6, 8].

Traditional Chinese medicine has been used to treat diseases, many effective components of traditional Chinese medicine are produced with strong biological activity after metabolism of intestinal flora and playing a therapeutic role [9, 10]. Puerarin, iso-flavone and other ingredients contained in *Huangta, Pueraria* and Douchi are commonly found in many prescriptions and health products. In vitro studies have shown that puerarin and iso-flavone can be metabolized into more effective daidzein and maorui iso-flavone by bacteria in the intestinal tract, which has a strong therapeutic effect [11]. The intestinal flora is closely related to the medicine and food taken cut contact [12–14]. The composition of traditional Chinese medicine is complex [15]. The intestinal flora in the body plays a vital role in the treatment of traditional Chinese medicine entering the body, and its corresponding effective components to maintain the balance of the number of intestinal flora in the body. Traditional Chinese medicines, such as rhubarb, thenardite, etc. contain irritating ingredients, which can inhibit the resident some bacteria in the gut. Xie et al. [16] found that *Rhizoma Coptidis* and berberine can significantly reduce the proportion of *Firmicutes* and *Bacteroidetes* in feces of high fat diet (HFD) mice in the total number of bacteria.

In the current treatment of diseases, traditional Chinese medicine has the advantages of small adverse reactions, no dependence and so on, which has gradually been accepted by the world. For functional constipation, colitis and other diseases, the use of traditional Chinese medicine has often achieve good results. Therefore, the demand for traditional Chinese medicine is increasing. Hence, the present study objective was mosapride used as a positive control, and diphenoxylate, a commonly used anti abdominal cathartic, combined with the qi stagnation and constipation rats caused by tail entrapment. In addition, self-developed traditional Chinese medicine decoction on constipation was used as the research object to explore the efficacy and the relationship between its efficacy and intestinal flora, to reveal its micro ecological mechanism of action. The development of the efficacy and function of traditional Chinese medicine provides certain theoretical basis to treatment of the disease.

## Materials and methods

### Animals and ethics statement

The Specific pathogen free (SPF) Wistar rats, male, were used for experiment purposes with body weight 110 ± 10 g. All animal tests were conducted in accordance with the NIH Publication No. 80-23 and approved by Fujian University of traditional Chinese medicine.

### Preparation of Chinese medicine

The traditional Chinese medicine “Li Qi Run Chang Fang” was prepared by the pharmacy of the second people’s Hospital Affiliated to Fujian University of traditional Chinese medicine. Drug composition: *Magnolia officinalis* 100 g, *Fructus aurantii* 100 g, hemp seed 150 g, plum seed 100 g, Fructus Trichosanthis 150 g, fried semen Raphani 100 g, bupleurum 90 g, paeony 120 g, tangerine peel 90 g, and mirabilite 30 g. When cooking, add eight times of water to soak for 0.5 h, then cook three times, the first, second and third time were 1.5 h, 1 h and 0.5 h, respectively, combine the supernatant and filter. The filtrate was concentrated to 500 ml under reduced pressure at 60–70 °C, and the concentration of the solution is 2.06 g/ml, which was sterilized and repacked, and stored in refrigerator at 4 °C.

### Constipation constipation model group (FC) rat model

This model was based on a FC rat model that made by intragastric administration of diphenoxylate, and combines the stagnation and constipation type caused by tail irritation: in addition to the blank control group, 10 rats in each model group are placed in the same cage, and the tail of the rats is entrapped with hemostatic forceps. The rats were stimulated for 30 min every time, 4 times a day, and the interval between the two stimulation was 3 h, a total of 14 days. Then, 10 mg/kg compound diphenoxylate was given to the stomach once a day, continuously for 7 days as a course of treatment. When the rats appeared irritable and fidgety, the first black stool excretion time was prolonged when they were fed with common diet, which was a successful model. Three rats were killed and their stomachs were taken to verify the success of the model.

### Study design

The 10 rats in the blank control group were fed SPF standard diet until the end of the experiment. 50 FC rats models were made according to the above method. After successful modeling, rats were randomly divided into FC model group (n = 10), low dose group (n = 10, DI), medium dose group (n = 10, ZH), high dose group (n = 10, GA) and Mosapride group (n = 10, MO). According to the dosage conversion of clinical application, rats in the low, middle and high dose groups were 5.15 g/(kg d), 10.3 g/(kg d) and 20.6 g/(kg d), respectively, while the solution was diluted to 4 ml/rat with physiological saline according to the weight of rats. According to the clinical dosage conversion, mosapride group was given 2 mg/(kg d) gavage of 4 ml/animal each time. The blank group and FC model control group were given 4 ml normal saline every time and gavage for 2 weeks.

### Bioinformatics and statistical analysis

Operational taxonomic unit (OTU) was generated by clustering clean tags. According to different similarity levels, OTUs of all sequences were divided, and the bio-information of OTUs above 97% similarity level was analyzed by Qiime software (version 1.8.0). The diversity analysis of single sample (alpha diversity) was reflect the richness and diversity of microbial community. The Qiime software (v1.8.0) was used to analyze the species richness and diversity of the intestinal flora. Use R software to draw and analyze PCoA graphics based on Bray–Curtis. Lefse analysis was used to analyze the correlation between the OTU of the first 20 absolute abundance samples and the gate-level annotation results through the spearman test and the Spearman test.

### DNA extraction and quantitative real time PCR (qRT-PCR) analysis

Extract DNA from stool from DNA stool kit. 338F 5′-GTACTCCTACGGGAGGCAGCA-3′ and 806R 5′-GTGGACTACHVGGGTWTCTAAT-3′ were used as primers to detect the copy number of 16srdna gene and calculate the total number of bacterial cells. Using total bacterial DNA as a template, PCR amplification was performed on the 16sv3-V4 region. Use RT-qPCR instrument (Bio-Rad CFX96™, BIO-RAD) for amplification. The reaction conditions were: pre denaturation at 94 °C for 5 min, 94 °C for 30 s, 55 °C for 30 s, and 72 °C for 30 s. After 30 cycles, 72 °C extended for 10 min, 4 °C insulation. After that, the purified product was connected to T-vector pmd19 vector and transformed into *E. coli* DH-α 5 sensitive cells. The plasmid DNA was extracted, and the qRT-PCR reaction was performed with plasmid standards of different concentrations as templates. Use the abscissa of the template copy number as the abscissa and the Ct value as the ordinate to draw the standard curve. Using sybrgreen as a fluorescent dye, a real-time quantitative PCR reaction was performed on stool samples to calculate the copy number. Sequencing was performed on the MiseqPE300 platform.

According to the regulations of cut-adapt, the original reading was filtered by mass under specific filtering conditions to obtain high-quality pure reading.

### Statistical analysis

Data shown are the means ± SD. T-test (prism 6.0) was used to analyze the data differences between the two groups. One way ANOVA (prism 6.0) was used to analyze the data difference more than two groups and the difference was significant (p < 0.05).

## Results

### Overall structural changes in microbiota composition

**Fig. 1:**
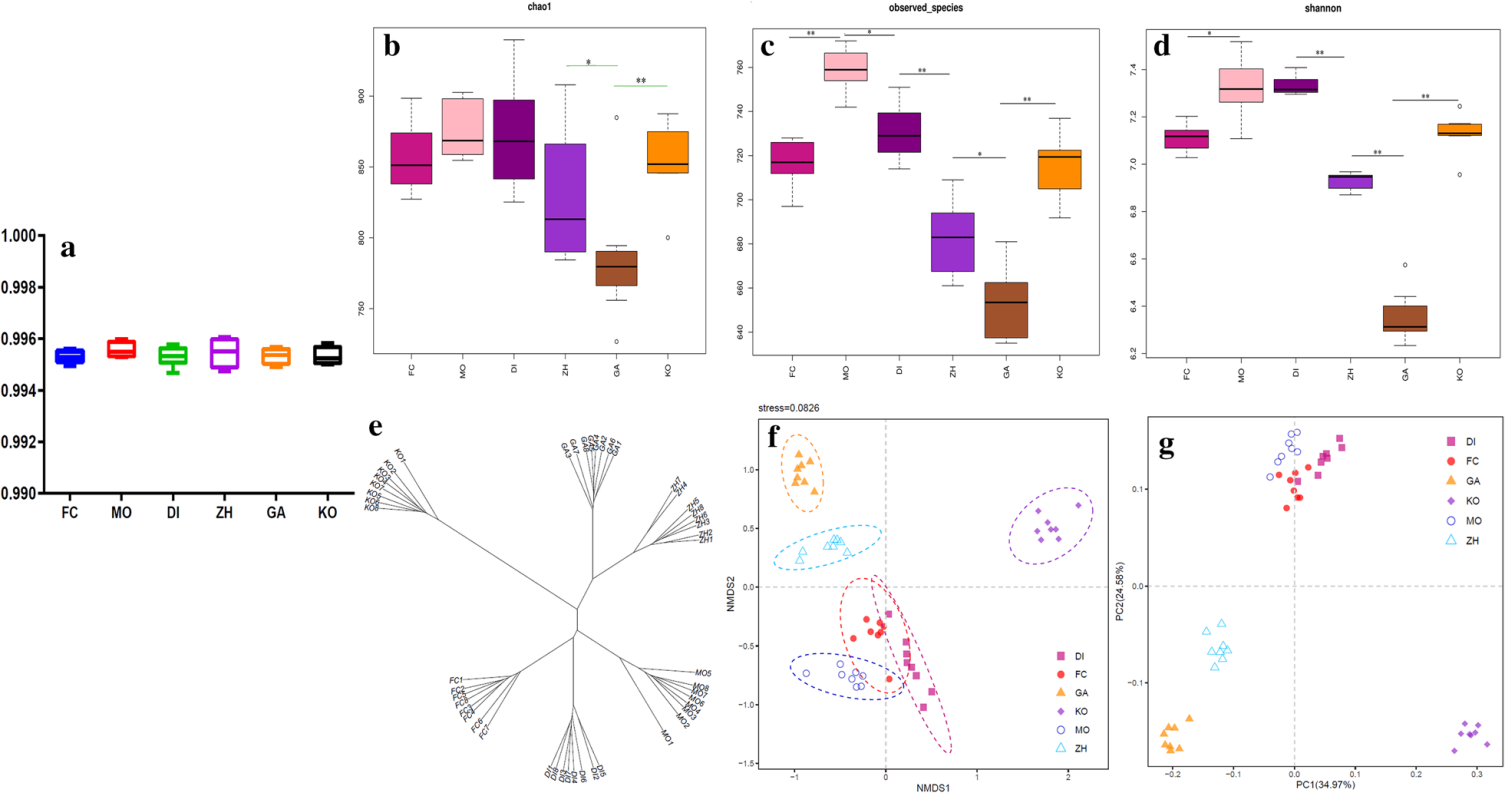
Changes in the diversity of the gut microbiota: **a–d** the goods coverage, Chao1 index, observed species and Shannon index of six groups; **e** Cluster based on analysis of hierarchical clustering using unweighted pair group method with arithmetic mean to study the similarity between different samples; **f** non-metric multidimensional scaling method (NMDS) is to simplify the research objects (samples or variables) in multidimensional space to low dimensional space for positioning, analysis and classification. The samples in the same group are all in a circle, which means that the differences between groups are not obvious, while the non-intersection between groups means that there are certain differences between groups; **g** PCoA plots based on Spearman distances are colored by time point. The significant differences between groups were calculated by analysis of similar (ANOSIM) tests. The data were analyzed by one-way ANOVA (*p < 0.05; **p < 0.01). *FC* constipation model group, *MO* mosapride group, *DI* low dose traditional Chinese medicine group, *ZH* middle dose traditional Chinese medicine group, *GH* high dose traditional Chinese medicine group, *KO* control group. n = 8/group

Dilution curve is used to reflect the rationality of sequencing data and indirectly the species richness. As shown in Additional file 1: Figures S1, S2, the curve gradually flattens, indicating that the amount of sequencing data is sufficient. At the same time, the good coverage of all experimental groups is greater than 99.0%, which indicates that the sequencing depth of the microbiome analysis is very deep and meets the requirements (Fig. 1a). α diversity analysis uses Chao and Shannon indexes to evaluate the richness and diversity of intestinal flora. As shown Fig. 1b, the richness of the bacterial population (Chao1 indices) in the GA group was significantly lower than that in the normal group (p < 0.01), and there was no significant difference in the species richness of intestinal flora between KO, FC, MO and DI Group compared with normal rats. The species richness of constipation model, mosapride group and low-medium-dose Chinese medicine group are similar to that of normal healthy rats, and there were no significant difference. The observed species indicates that the number of OTU actually observed with the increase in sequencing depth. As shown Fig. 1c, the number of OTU actually observed in the MO group was the largest, which is significantly higher than that in the FC (p < 0.01) and the DI group (p < 0.05). The low-medium-high-dose Chinese medicine group showed a gradually decreasing trend (p < 0.05). From Fig. 1d, the Shannon index of MO and DI group was significantly higher than that of normal group (p < 0.05), which were indicates that the diversity of intestinal flora of mosapride and DI group was more than that of healthy rats, while the intestinal diversity of constipation group was not significantly different from that of healthy rats. The intestinal diversity of GA group was significantly lower than that of normal control group (p < 0.01) and ZH group (p < 0.01), which were indicating that Chinese medicine at a high dose reduced the intestinal flora diversity.

In order to further study, the similarity or difference of the composition of the intestinal flora of the sample, cluster, Non-metric multidimensional scaling method (NMDS) and principal co-ordinates analysis (PCoA) were performed. Cluster analysis uses tree structure to describe and compare similarities between multiple samples. It can be clearly seen from Fig. 1e that the microbiological composition of the samples in the group is similar, and the samples of the same treatment group was gathered together, indicating that the differences between the groups was small and the sample repeatability good. It can be seen from NMDS and PCoA analysis (Fig. 1f, g) that the microbial composition of the normal control group was different from that of DI, ZH and GA groups, indicating that the intake of low, medium and high doses of traditional Chinese medicine changes the composition of the whole intestinal flora In addition, it can be found that the differences between FC group and MO and DI groups are not obvious, and the composition of the flora tends to be the same, indicating that the intestinal flora composition of the constipation group, the mosapride group and the low-dose Chinese medicine group similar.

### Classification based comparison of phylum and genus levels

**Fig. 2:**
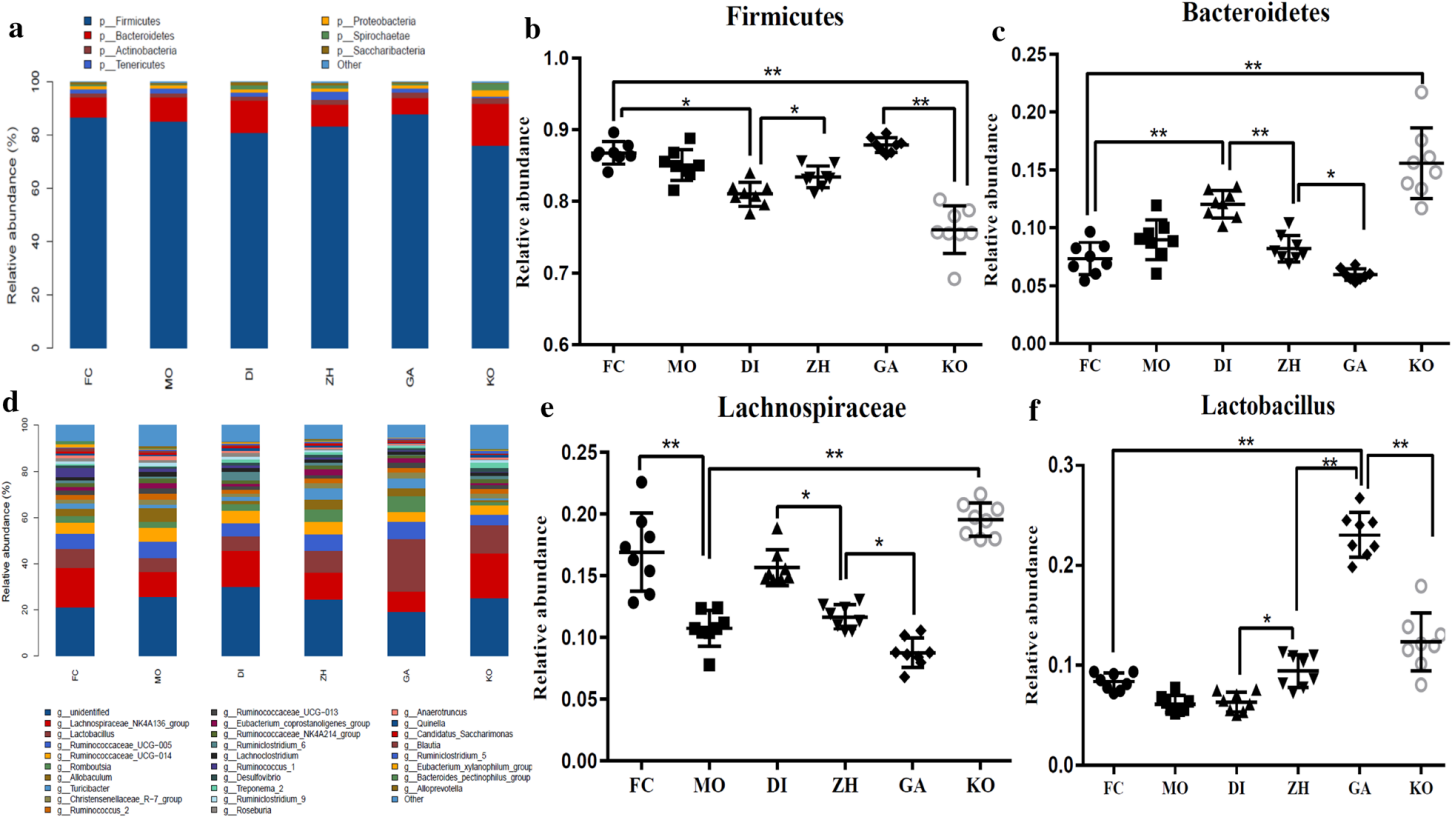
Composition of the gut microbiota: **a** the relative contribution of the top 8 phyla in each group; **b** relative abundance of *Firmicutes* in six groups, the data were analyzed by one-way ANOVA (*p < 0.05; **p < 0.01); **c** Relative abundance of *Bacteroides* in six groups, the data were analyzed by one-way ANOVA (*p < 0.05; **p < 0.01); **d** the relative contribution of the top 30 genera in each group; **e** relative abundance of *Lachnospiraceae* in six groups, the data were analyzed by one-way ANOVA (*p < 0.05; **p < 0.01); **f** relative abundance of *Lactobacillus* in six groups, the data were analyzed by one-way ANOVA (*p < 0.05; **p < 0.01)

From the phylum-level analysis (Fig. 2a), we could clearly found that 90% of the intestinal microorganisms in six groups were mainly composed of *Firmicutes* and *Bacteroides*. The abundances of *Firmicutes* in KO, FC, MO, DI, ZH and GA groups were 76.0%, 86.7%, 85.1%, 81.0%, 83.4% and 87.8%, respectively. The relative abundances of *Bacteroides* in KO, FC, MO, DI, ZH and GA groups were 15.7%, 7.3%, 9.0%, 12.0%, 8.2% and 6.0%, respectively. It can be seen from Fig. 2b that the relative abundance of *Firmicutes* in the constipation model group, MO group and GA group were significantly higher than that in the control group (p < 0.01), and there were no significant difference between the three groups. Meanwhile, the abundance of *Firmicutes* in the DI group was significantly lower than that in the middle dose ZH and constipation group (p < 0.05). As can be seen from Fig. 2c, the relative abundance of *Bacteroidetes* in the FC, MO group and the different doses of the Chinese medicine group was significantly lower than the control group (p < 0.01). At the same time, the relative abundance of *Bacteroidetes* in the DI group was significantly higher than that in the FC group. At the same time, it showed a gradual decrease with the rising the dose of Chinese medicine (p < 0.05).

We could clearly found that the intestinal microorganisms in six groups were mainly composed of *Lachnospiraceae, Lactobacillus* and *Ruminococcaceae* (Fig. 2d)*.* The abundances of *Lachnospiraceae* in KO, FC, MO, DI, ZH and GA groups were 19.5%, 16.9%, 10.8%, 15.7%, 11.7% and 8.8%, respectively. The abundances of *Lactobacillus* in KO, FC, MO, DI, ZH and GA groups were 12.3%, 8.3%, 6.1%, 6.3%, 9.4% and 23.1%, respectively. The abundances of *Ruminococcaceae* in KO, FC, MO, DI, ZH and GA groups were 8.3%, 11.4%, 12.9%, 11.0%, 12.5% and 11.7%. We found that there was no significant difference in the relative abundance of *Ruminococcaceae* among the six groups. The abundance of *Lachnospiraceae* decreased with the increasing of the dosage of Chinese medicine (p < 0.05), at the same time, the intake of Mosapride significantly reduced the relative abundance of *Lachnospiraceae* (p < 0.01) (Fig. 2e). The abundance of *Lactobacillus* increased with the increasing of the dosage of Chinese medicine, and the GA group was significantly higher than other group (p < 0.01) (Fig. 2f). The results indicated that the abundance of *Lactobacillus* in the FC group was significantly lower than KO (p < 0.05). *Lactobacillus* has a significant role in promoting intestinal peristalsis, so we speculate that the role of traditional Chinese medicine in the treatment of constipation may be related to promoting the proliferation of probiotics.

### The differences in the dominant members of the microbiota

**Fig. 3:**
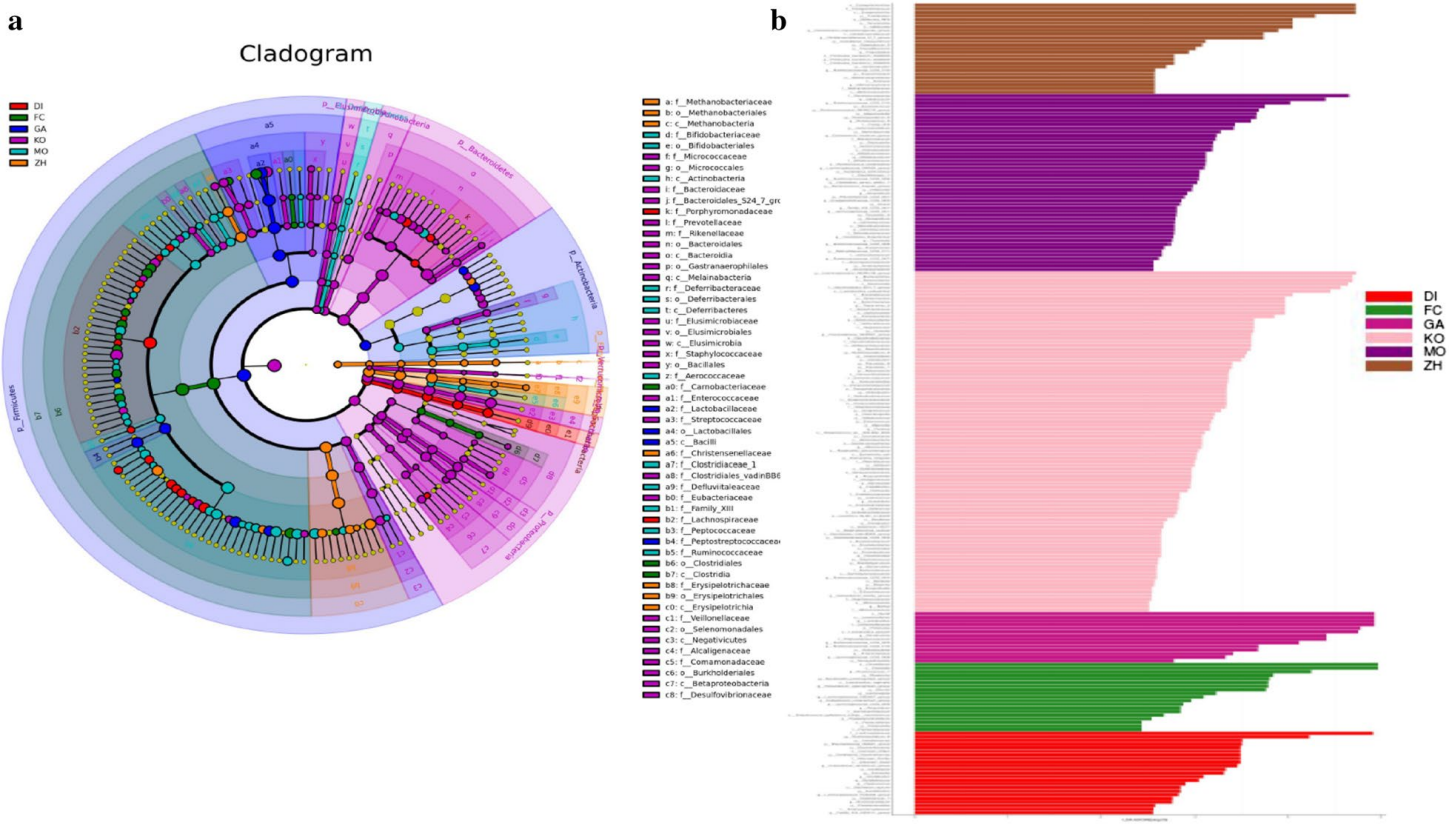
Major differential microbial species: **a–b** Taxonomic cladogram obtained from LEfSe at six groups. Biomarker taxa are highlighted with colored circles and shaded areas. Each circle’s diameter reflects the abundance of those taxa in the community

In order to verify and further determine the LEfSe was used to identify the specific phylotypes responding to FC and DI, ZH, GA groups. As shown Fig. 3a, b, the main differential microbial species between the FC and other groups were *Carnobacteriaceae* and *Clostridiales*, the main differential microbial species between the DI group and other groups were *Porphyromonadaceae* and *Lachnospiraceae*, the main differential microbial species between the ZH group and other groups were *Methylobacteriaceae*, *Christensenellaceae* and *Erysipelotrichaceae*, the main differential microbial species between the GA group and other groups were *Lactobacillaceae*, *Bacilli* and *Peptostreptococcaceae*, the main differential microbial species between the MO group and other groups were *Bifidobacteriaceae*, *Actinomycetaceae*, *Deferribacteraceae*, *Aerococcaceae*, *Clostridiaceae*, *Peptococcaceae* and *Ruminococcaceae*.

### Network relationship and functional prediction analysis

**Fig. 4:**
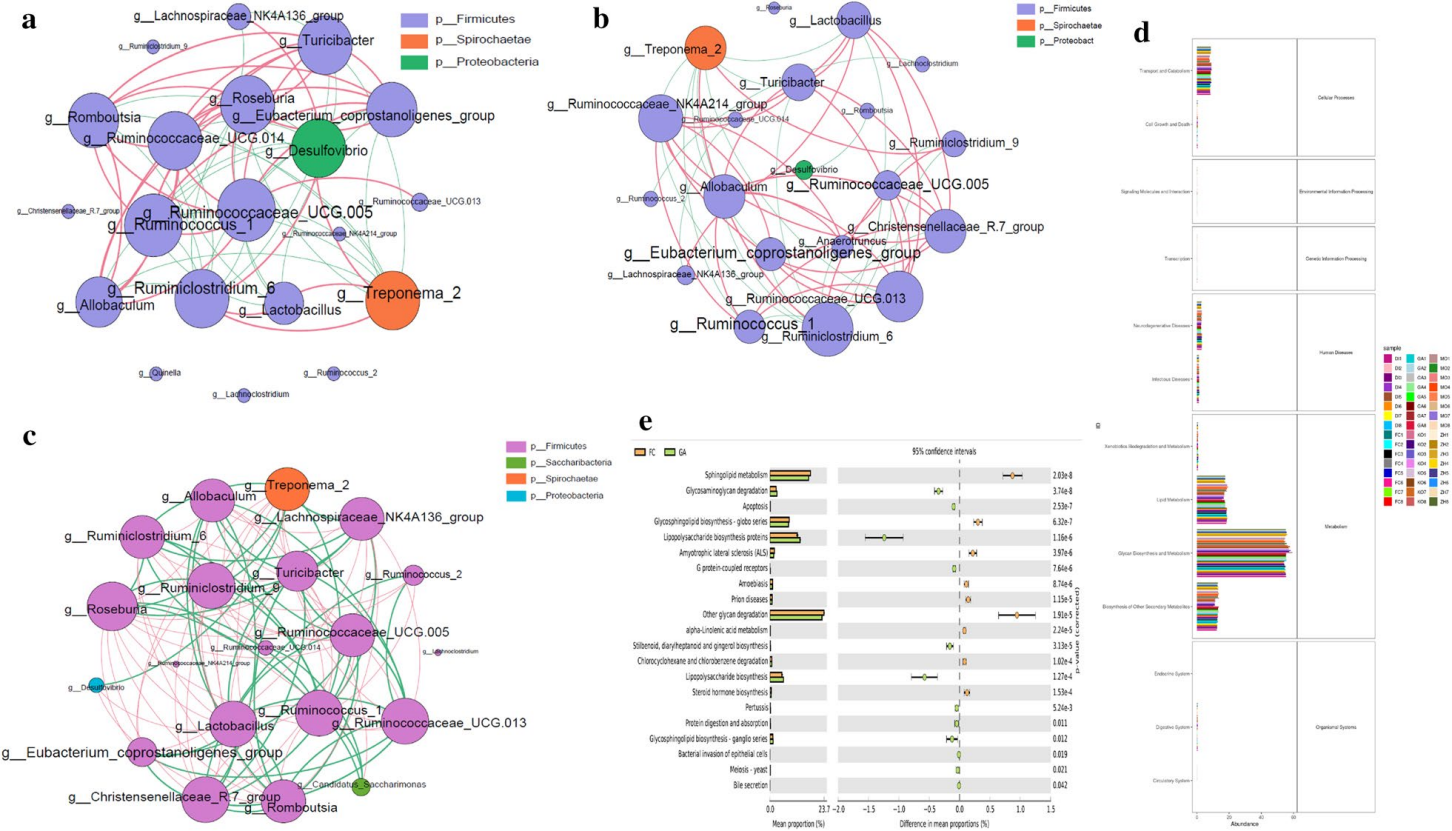
Co-occurrence network and functional prediction analysis: **a** co-occurrence network of the control group KO; **b** co-occurrence network of the FC group; **c** co-occurrence network of supplementation with high-dose dose of Chinese medicine. Blue indicates positive correlation, red indicates negative correlation. The numbers of nodes and links indicate the tightness of the connection between the bacteria and others, indicating the strength of the symbiotic relationship; **d** functional gene abundance for the intestinal microbiota; **e** analysis of gene difference between FC and GA groups

Faust et al. [17] proposed a network reasoning analysis method based on the relationship between microbial members. The purpose of this analysis was to explore and analyze the interaction patterns between members of the gut microbial community in samples from different treatment groups. The numbers of the nodes and links were counted through statistical network symbiosis, it can be seen from Fig. 4a that the dominant symbiotic dominant flora in the KO group is *Firmicutes, Spirochaetae* and *Proteobacteria*. At the same time, the genus level of *Firmicutes*, the dominant symbiotic flora includes *Turibacharacter, Roseburia, Eubacterium,* and other bacteria*.* In addition, 7 symbiotic relationships between *Lactobacillus* and other bacteria, 5 positive and 2 negative ones. From Fig. 4b that the dominant symbiotic dominant flora in the FC group is *Firmicutes* and *Spirochaetae*. At the genus level of *Firmicutes*, the dominant symbiotic flora includes *Turicibacter, Eubacterium,* and *Treponema.* There are 7 symbiotic relationships between *Lactobacillus* and other bacteria, 2 positive and 5 negative ones. The above shows that in constipation group FC was significantly reduce the symbiotic relationship between *Proteobacteria* and other intestinal bacteria, increase symbiosis of *Firmicutes* and other flora, but it does not change and affect the symbiotic flora of *Spirochaetae*. Compared with control group KO, the negative symbiotic relationship of Lactobacillus was increased in the constipation group. From Fig. 4c that the dominant symbiotic flora in the GA group was *Firmicutes* and *Spirochaetae*. At the genus level of *Firmicutes*, the dominant symbiotic flora includes *Lactobacillus, Turicibacter, Eubacterium* and other bacteria*.* There are 24 symbiotic relationships between *Lactobacillus* and other bacteria, 14 positive ones and 13 negative ones. In this study, we found that a certain dose of Chinese medicine significantly increased the symbiotic relationship between *Lactobacillus* and other intestinal flora, making *Lactobacillus* from the edge of the symbiotic relationship to the dominant dominant flora.

Changes in the symbiotic relationship of the gut microbiota often also indicate functional changes; thus, functional prediction analysis was also performed. From Fig. 4d, it can be seen that the distribution of functional genes in these six groups is mainly concentrated in glycan biosynthesis and betabolism, followed by Lipid metabolism, biosynthesis of other secondary metabolites and transport and catabolism. At the same time, the Fig. 4e also indicted that the expression of glycosaminoglycan degradation, apoptosis, G protein-coupled receptors, stilbenoid, diarylheptanoid and gingerol biosynthesis, protein digestion and absorption, glycosphingolipid biosynthesis—ganglio series and bill secretion were significantly up-regulated by Chinese medicine compared with FC group, significant down-regulation of glycosphingolipid biosynthesis—globo series, amyotrophic lateral sclerosis (ALS), amoebiasis, prion diseases, alpha-linolenic acid metabolism, chlorocyclohexane and chlorobenzene degradation and steroid hormone biosynthesis.

### qRT-PCR analysis

The total copy number range of 16S rDNA gene of bacteria was from 8.4 × 10^7^ to 8.2 × 10^8^, 2.1 × 10^8^ to 1.1 × 10^10^, 1.4 × 10^10^ to 9.0 × 10^13^, 4.1 × 10^10^ to 5.9 × 10^12^, 3.2 × 10^6^ to 1.4 × 10^9^, 2.5 × 10^6^ to 1.3 × 10^8^ copies per gram of tissue content in the KO, FC, MO, DI, ZH and GA samples, respectively. We found that the total copy number of bacteria in the FC group was not significantly different from that in the control group, and that in the MO group and the DI group was significantly higher than that KO (p < 0.05). The difference between the medium dose traditional Chinese medicine group and the control group was not significant, while the copy number of bacteria in the high-dose group was significantly lower than that KO (Table 1).

**Table 1:** Total copies of intestinal bacteria in each group

Total bacteria (copy number/g)	FC	MO	DI	ZH	GA	KO
1	1,482,131,000	14,424,390,000	1.30734E+11	877,964,800	2,532,737	821,453,400
2	530,056,400	66,255,020,000	5.94403E+12	1,375,961,000	45,190,660	84,881,970
3	476,350,900	14,133,200,000	4.09941E E+12	65,151,450	69,905,180	84,399,230
4	3,238,729,000	1.35541E+12	41,278,390,000	16,213,720	144,515,900	311,785,000
5	216,104,600	2.33782E+11	52,287,600,000	17,307,860	84,531,920	178,919,400
6	241,080,800	9.06385E+13	54,849,440,000	10,236,970	107,940,800	168,030,700
7	4,593,153,000	65,143,480,000	5.61591E+11	3,292,147	120,057,200	820,202,500
8	11,431,060,000	63,403,100,000	1.96913E+11	5,663,928	133,493,100	490,321,300

*FC* constipation model group, *MO* mosapride group was given 2 mg/(kg d) gavage, *DI* low dose group with 5.15 g/(kg d), *ZH* medium dose group with 10.3 g/(kg d), *GA* high dose group with 20.6 g/(kg d) doses, *KO* control group

## Discussion

Gut microbes were closely related to human health, which help regulate the metabolism of the host and the development of the immune system [18]. Numerous studies have shown that intestinal microbes significantly associated with many diseases of the human body, such as intestinal inflammation, obesity, diabetes, and tumors [19, 20]. At the same time, it was found that constipation was closely related to intestinal flora. On the one hand, constipation reduces the number of beneficial bacteria in the intestine and increases the number of pathogenic bacteria or conditioned pathogens; when the structure of the flora is disordered, conditioned pathogens and pathogens can cause the displacement of intestinal bacteria, and then lead to the release of a large number of inflammatory factors, which can further aggravate the symptoms of constipation. Therefore, in the process of constipation, intestinal flora disorder was often accompanied by some inflammatory reactions [13].

16S rRNA high-throughput sequencing technology has been used to explored the fecal flora in constipation and healthy children and adults [21]. Khalif et al. [22] studied 57 adult patients with functional constipation, and found that the content of *Bifidobacteria* and *Lactobacillus* decreased significantly, but the potential pathogenic bacteria or fungi increased. The *Firmicutes*/*Bacteroides* value of children with chronic constipation is higher than that of healthy children, the number of *Prevotella* is significantly reduced, and the number of *Bifidobacteria* and *Fusobacteria* is not significantly reduced [23]. This result is consistent with that of our study, where we found that the relative abundance of *Firmicutes* in the intestine of constipation model rats was significantly higher than normal group, and the relative abundance of *Bacteroides* decreased. Zhu et al. [23] studied the fecal microbial composition of 8 children with constipation and 14 healthy children. From the level of phylum, the proportion of *Bacteroides* in healthy people were the first dominant group. It is the main part of the intestinal flora. The proportion of constipation population was significantly different from that of healthy people, with the proportion of *Firmicutes* being the first dominant flora.

After observation of different doses of traditional Chinese medicine to rats for a period of time, through 16S rRNA sequencing results, we found that different doses of traditional Chinese medicine significantly reduced the relative abundance of *Lachnospiraceae*, and greatly increased the relative abundance of *Lactobacillus*. As a probiotic, *Lactobacillus* can promote intestinal peristalsis, it can promote intestinal peristalsis. Therefore, we speculated whether the reason why this traditional Chinese medicine plays a role in reducing constipation was related to promoting the relative abundance of *Lactobacillus*. According to relevant literature research [24], we found that the depth of colonic crypt in constipated rats decreased, there were a lot of inflammatory reactions, goblet cells were lost, and intestinal crypt had the function of protecting intestinal stem cells and preventing intestinal cells from being damaged. At the same time, the goblet cells secrete mucin, which were forms the mucus barrier, provides a place for the symbiotic bacteria in the host, prevents the colonization of pathogenic microorganism. At the same time, some studies have found that probiotics such as *Lactobacillus* can enhance intestinal epithelial barrier function and promote intestinal growth in mice with ulcerative colitis, thereby reducing the severity of colon damage [24], so we speculate that the reduction of constipation may be closely related to the significant increase of *Lactobacillus* in the feces, which can protect the colon tissue. Chen et al. [25] found *Lactobacillus paracasei* could increase short-chain fatty acid levels, reduced fecal pH value, enhanced the thickness of the colonic mucosa, and increased the number of mucin-producing goblet cells and interstitial cells of Cajal. Thus, Therefore, they speculate that *Lactobacillus paracasei* may reduce the constipation caused by loperamide and improve the gastrointestinal function through the above methods and related mechanisms.

At the same time, *Lactobacillus* can promote intestinal peristalsis, metabolize short chain fatty acids and other beneficial products, and promote intestinal health. By GC/MS and targeted metabolites, *Lactobacillus casei* strains were found to significantly improve defecation frequency, and 14 kinds of nonvolatile fecal metabolites, as possible constipation related metabolites, were regulated by *Lactobacillus casei* [8]. In the next step of this study, we would like to build the constipation model of germ-free mice, transplant *Lactobacillus* into aseptic mice to explore the specific mechanism of improving constipation, and combine 16S rRNA sequencing technology with metabolome to explore the mechanism.

## Conclusion

In general, we found that *Firmicutes* in constipation group was significantly higher. The intake of different doses of traditional Chinese medicine significantly increased the relative abundance of *Lactobacillus*, enhanced the symbiotic relationships of *Lactobacillus* with other intestinal flora, reduced the production of constipation. It provided a theoretical basis for the treatment and mechanism of constipation.

## Supplementary information

**Additional file 1: Figure S1.** Multy samples rarefaction curves for each group.** Figure S2.** Number of samples sequenced for each group.
