# Supplementary material for: Formulation of traditional Chinese medicine and its application on intestinal flora of constipated rats
Source: Microb Cell Fact. 2020 Nov 18;19:212. doi: 10.1186/s12934-020-01473-3 (PMC7672833; doi:10.1186/s12934-020-01473-3)
Supplement: Supplementary file 1 — Additional file 1: Figure S1. Multy samples rarefaction curves for each group. Figure S2. Number of samples sequenced for each group. [file 12934_2020_1473_MOESM1_ESM.docx]

**Formulation of traditional Chinese medicine and its application on intestinal flora of constipated rats**

**Sihan Li^1,2,†^, Youcheng He^1,†^, Haiou Zhang^1^, Rong Zheng^1^, Ruoying Xu^1^, Qihong Liu^1^, Shuihua Tang^1^, Xiao Ke^1,*^ and Minghan Huang^1,*^**

^1^Department of Gastroenterology, the second people's Hospital affiliated to Fujian University of traditional Chinese Medicine, Fuzhou 353003, China

^2^School of Basic Medical Sciences, Guangzhou University of Chinese Medicine, Guangzhou, China

**^†^**These two authors contributed equally to this work;

***Corresponding author:**

**Prof. Minghan Huang,**

Email: [huangminghan2010@163.com](mailto:huangminghan2010@163.com)

**Prof.** **Xiao Ke,**

Email: drkxkx@163.com

**Additional Figure captions:**

**Fig. 1S.** Multy samples rarefaction curves for each group.

**Fig. 2S.** Number of samples sequenced for each group.

**Fig. 1S.**

**Fig. 2S.**
